# Poly[(acetato-κ^2^ *O*,*O*′)aqua­(μ_4_-1*H*-benzimidazole-5,6-dicarboxyl­ato-κ^6^ *N* ^3^:*O* ^5^,*O* ^5′^:*O* ^5^,*O* ^6^:*O* ^6′^)cerium(III)]

**DOI:** 10.1107/S1600536812017503

**Published:** 2012-04-25

**Authors:** Jinhua Chen, Yuezhu Wang, Chun Zheng, Yifan Luo

**Affiliations:** a School of Chemistry and Environment, South China Normal University, Guangzhou 510006, People’s Republic of China

## Abstract

In the title compound, [Ce(C_9_H_4_N_2_O_4_)(C_2_H_3_O_2_)(H_2_O)]_*n*_, the Ce^III^ ion is coordinated by five O atoms and one N atom from four 1*H*-benzimidazole-5,6-dicarboxyl­ato (*L*) ligands and by two O atoms from an acetate ligand and one aqua ligand, forming a slightly distorted tricapped trigonal–prismatic geometry. The *L* ligands are bridging, forming a layered polymer parallel to (010). In the crystal, O—H⋯O and N—H⋯O hydrogen bonds connect the polymer layers into a three-dimensional network.

## Related literature

For background to 1*H*-benzimidazole-5,6-dicarboxyl­ate complexes and for related structures, see: Gao *et al.* (2008 ▶); Yao *et al.* (2008 ▶); Song, Wang, Hu *et al.* (2009 ▶); Song, Wang, Li *et al.* (2009 ▶).
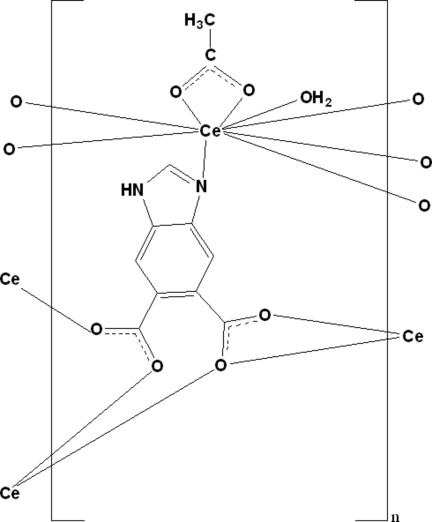


## Experimental

### 

#### Crystal data

[Ce(C_9_H_4_N_2_O_4_)(C_2_H_3_O_2_)(H_2_O)]
*M* *_r_* = 421.32
Triclinic, 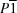

*a* = 7.4577 (15) Å
*b* = 9.0399 (19) Å
*c* = 9.792 (2) Å
α = 86.895 (2)°
β = 86.510 (2)°
γ = 84.707 (2)°
*V* = 655.3 (2) Å^3^
*Z* = 2
Mo *K*α radiation
μ = 3.51 mm^−1^
*T* = 293 K
0.17 × 0.13 × 0.11 mm

#### Data collection

Bruker APEXII CCD diffractometer
Absorption correction: multi-scan (*SADABS*; Bruker, 2005 ▶) *T*_min_ = 0.584, *T*_max_ = 0.680
3390 measured reflections
2366 independent reflections
2194 reflections with *I* > 2σ(*I*)
*R*_int_ = 0.016

#### Refinement

*R*[*F* ^2^ > 2σ(*F* ^2^)] = 0.023
*wR*(*F* ^2^) = 0.054
*S* = 1.05
2321 reflections
203 parameters
130 restraints
H atoms treated by a mixture of independent and constrained refinement
Δρ_max_ = 0.74 e Å^−3^
Δρ_min_ = −0.59 e Å^−3^

### 

Data collection: *APEX2* (Bruker, 2005 ▶); cell refinement: *SAINT* (Bruker, 2005 ▶); data reduction: *SAINT*; program(s) used to solve structure: *SHELXS97* (Sheldrick, 2008 ▶); program(s) used to refine structure: *SHELXL97* (Sheldrick, 2008 ▶); molecular graphics: *ORTEP-3* (Farrugia, 1997 ▶); software used to prepare material for publication: *SHELXL97* and *PLATON* (Spek, 2009 ▶).

## Supplementary Material

Crystal structure: contains datablock(s) I, global. DOI: 10.1107/S1600536812017503/lh5440sup1.cif

Structure factors: contains datablock(s) I. DOI: 10.1107/S1600536812017503/lh5440Isup2.hkl

Additional supplementary materials:  crystallographic information; 3D view; checkCIF report

## Figures and Tables

**Table 1:** Hydrogen-bond geometry (Å, °)

*D*—H⋯*A*	*D*—H	H⋯*A*	*D*⋯*A*	*D*—H⋯*A*
N2—H2⋯O5^i^	0.86 (3)	1.93 (3)	2.716 (4)	153 (4)
O7—H7*A*⋯O3^ii^	0.84 (4)	2.05 (4)	2.850 (4)	158 (3)
O7—H7*B*⋯O6^iii^	0.83 (3)	1.95 (3)	2.777 (4)	176 (6)

Symmetry codes: (i) 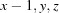; (ii) 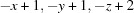; (iii) 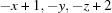.

**Fig. 1.**
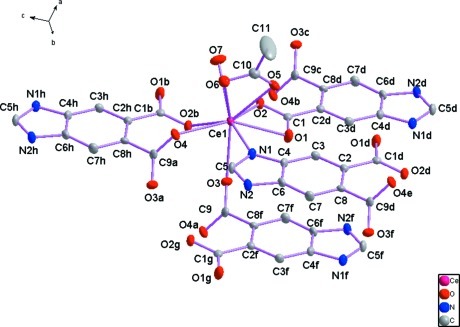
The asymmetric unit of the title compound, together with some symmetry related atoms to complete the coordination of the CeIII ion. Displacement ellipsoids are shown at the 50% probability level. [Symmetry codes: (a) = -x, 1-y, 2-z; (b )= 1-x, 1-y, 2-z; (c) = 1+x, y, z; (d) = 1-x, 1-y, 1-z; (e) = x, y, -1+z; (f) = -x, 1-y, 1-z; (g) = -1+x, y, z; (h) = x, y, 1+z].

**Fig. 2.**
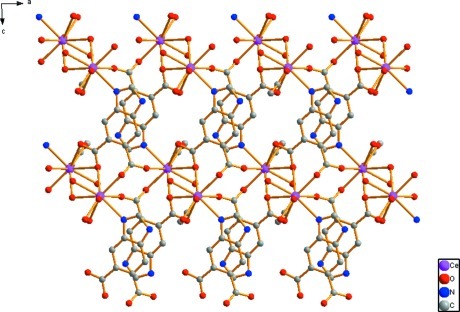
Part of the crystal structure showing polymeric layers parallel to the ac plane. H atoms are not showm.

## supplementary crystallographic information

### Comment

1*H*-Benzimidazole-5,6-dicarboxylic acid (H_2_L) acts as a multidentate ligand forming coordination polymers (Gao *et al.*, 2008; Yao *et al.*, 2008; Song, Wang, Hu *et al.*, 2009; Song, Wang, Li *et al.*, 2009). Herein, we report the Ce(III) complex of H_2_L. The asymmetric unit of the title compound with additional symmetry related atoms is shown in Fig. 1. The Ce^III^ ion is coordinated by five O atoms and one N atom originating from four 1*H*-benzimidazole-5,6-dicarboxylate (L) ligands along with two O atoms from an acetate ligand and one aqua ligand forming a slightly distorted tricapped trigonal-prismatic geometry. The L ligands act as bridging forming a layered polymer parallel to (010) (Fig. 2). In the crystal, intermolecular O—H···O and N—H···O hydrogen bonds connect the polymer layers into a three-dimensional network.

### Experimental

A mixture of Ce_2_O_3_ (0.4 mmol), H_2_L (0.4 mmol), acetic acid (0.4 mmol) and water (13 ml) was added in a 25 ml teflon-lined stainless container, which was heated at 453 K for 3 days. After cooling to room temperature, colorless crystals were recovered by filtration.

### Refinement

H atoms bonded to N and O atoms were located in difference Fourier maps and refined with isotropic displacement parameters. H atoms bonded to C atoms were placed in calculated positions and included in the refinement using a riding-model approximation [C–H = 0.93-0.96 with *U*_iso_(H) = 1.2*U*_eq_(C) or 1.5*U*_eq_(C_methyl_). The 'SIMU 0.5 0.5 3.8 $C' instruction in SHELXL (Sheldrick, 2008) was used to restrain the anisotropic displacement parameters of the C atoms.

### Crystal data

**Table:**

[Ce(C_9_H_4_N_2_O_4_)(C_2_H_3_O_2_)(H_2_O)]	*Z* = 2
*M**_r_* = 421.32	*F*(000) = 406
Triclinic, *P*1	*D*_x_ = 2.135 Mg m^−^^3^
Hall symbol: -P 1	Mo *K*α radiation, λ = 0.71073 Å
*a* = 7.4577 (15) Å	Cell parameters from 2613 reflections
*b* = 9.0399 (19) Å	θ = 2.1–25.2°
*c* = 9.792 (2) Å	µ = 3.51 mm^−^^1^
α = 86.895 (2)°	*T* = 293 K
β = 86.510 (2)°	Block, colorless
γ = 84.707 (2)°	0.17 × 0.13 × 0.11 mm
*V* = 655.3 (2) Å^3^	

### Data collection

**Table:**

Bruker APEXII CCD diffractometer	2366 independent reflections
Radiation source: fine-focus sealed tube	2194 reflections with *I* > 2σ(*I*)
Graphite monochromator	*R*_int_ = 0.016
φ and ω scans	θ_max_ = 25.2°, θ_min_ = 2.1°
Absorption correction: multi-scan (*SADABS*; Bruker, 2005)	*h* = −8→8
*T*_min_ = 0.584, *T*_max_ = 0.680	*k* = −10→7
3390 measured reflections	*l* = −11→10

### Refinement

**Table:**

Refinement on *F*^2^	Primary atom site location: structure-invariant direct methods
Least-squares matrix: full	Secondary atom site location: difference Fourier map
*R*[*F*^2^ > 2σ(*F*^2^)] = 0.023	Hydrogen site location: inferred from neighbouring sites
*wR*(*F*^2^) = 0.054	H atoms treated by a mixture of independent and constrained refinement
*S* = 1.05	*w* = 1/[σ^2^(*F*_o_^2^) + (0.0217*P*)^2^ + 1.2938*P*] where *P* = (*F*_o_^2^ + 2*F*_c_^2^)/3
2321 reflections	(Δ/σ)_max_ = 0.004
203 parameters	Δρ_max_ = 0.74 e Å^−^^3^
130 restraints	Δρ_min_ = −0.59 e Å^−^^3^

### Special details

**Table:**

Geometry. All e.s.d.'s (except the e.s.d. in the dihedral angle between two l.s. planes) are estimated using the full covariance matrix. The cell e.s.d.'s are taken into account individually in the estimation of e.s.d.'s in distances, angles and torsion angles; correlations between e.s.d.'s in cell parameters are only used when they are defined by crystal symmetry. An approximate (isotropic) treatment of cell e.s.d.'s is used for estimating e.s.d.'s involving l.s. planes.
Refinement. Refinement of *F*^2^ against ALL reflections. The weighted *R*-factor *wR* and goodness of fit *S* are based on *F*^2^, conventional *R*-factors *R* are based on *F*, with *F* set to zero for negative *F*^2^. The threshold expression of *F*^2^ > σ(*F*^2^) is used only for calculating *R*-factors(gt) *etc*. and is not relevant to the choice of reflections for refinement. *R*-factors based on *F*^2^ are statistically about twice as large as those based on *F*, and *R*- factors based on ALL data will be even larger.The number of independent reflections and the number of reflections used in the refinement are not the same,because we used 'omit -3 50' to enhance the '_diffrn_measured_fraction_theta_full'.

### Fractional atomic coordinates and isotropic or equivalent isotropic displacement parameters (Å^2^)

**Table:**

	*x*	*y*	*z*	*U*_iso_*/*U*_eq_	
Ce1	0.35253 (3)	0.35579 (2)	0.893484 (19)	0.01339 (8)	
O6	0.3692 (4)	0.0660 (3)	0.8693 (3)	0.0242 (6)	
O5	0.5196 (3)	0.1955 (3)	0.7120 (3)	0.0224 (6)	
O3	0.1354 (3)	0.5879 (3)	0.8893 (3)	0.0198 (6)	
O4	0.1202 (3)	0.2775 (3)	1.0582 (2)	0.0181 (5)	
O7	0.6258 (4)	0.2197 (3)	1.0107 (3)	0.0277 (7)	
O1	0.4775 (3)	0.5295 (3)	0.7017 (3)	0.0235 (6)	
O2	0.6399 (3)	0.5169 (3)	0.8819 (2)	0.0169 (5)	
N1	0.1240 (4)	0.3026 (3)	0.7095 (3)	0.0185 (7)	
N2	−0.1340 (4)	0.2436 (4)	0.6341 (3)	0.0199 (7)	
C4	0.1286 (5)	0.3230 (4)	0.5663 (3)	0.0152 (7)	
C5	−0.0343 (5)	0.2557 (4)	0.7418 (4)	0.0189 (8)	
H5	−0.0745	0.2328	0.8315	0.023*	
C6	−0.0331 (5)	0.2865 (4)	0.5188 (4)	0.0157 (7)	
C7	−0.0694 (5)	0.3027 (4)	0.3812 (4)	0.0175 (8)	
H7	−0.1804	0.2823	0.3516	0.021*	
C3	0.2613 (5)	0.3736 (4)	0.4746 (4)	0.0147 (7)	
H3	0.3695	0.3983	0.5059	0.018*	
C2	0.2310 (5)	0.3871 (4)	0.3353 (3)	0.0130 (7)	
C8	0.0645 (5)	0.3502 (4)	0.2889 (4)	0.0141 (7)	
C10	0.4720 (5)	0.0728 (4)	0.7632 (4)	0.0235 (9)	
C1	0.6200 (5)	0.5515 (4)	0.7549 (3)	0.0129 (7)	
C9	−0.0166 (5)	0.6502 (4)	0.8595 (3)	0.0139 (7)	
C11	0.5451 (11)	−0.0663 (6)	0.6954 (7)	0.084 (3)	
H11A	0.6391	−0.1166	0.7478	0.126*	
H11B	0.5930	−0.0413	0.6047	0.126*	
H11C	0.4501	−0.1302	0.6901	0.126*	
H7B	0.629 (6)	0.133 (3)	1.043 (5)	0.037 (14)*	
H7A	0.717 (5)	0.259 (4)	1.032 (5)	0.045 (15)*	
H2	−0.239 (3)	0.212 (5)	0.635 (5)	0.045 (15)*	

### Atomic displacement parameters (Å^2^)

**Table:**

	*U*^11^	*U*^22^	*U*^33^	*U*^12^	*U*^13^	*U*^23^
Ce1	0.01261 (12)	0.01670 (12)	0.01084 (11)	−0.00151 (8)	0.00020 (7)	−0.00120 (7)
O6	0.0253 (15)	0.0189 (14)	0.0274 (16)	−0.0020 (11)	0.0036 (12)	0.0014 (11)
O5	0.0166 (14)	0.0236 (15)	0.0257 (15)	0.0007 (11)	0.0058 (11)	−0.0019 (11)
O3	0.0172 (13)	0.0229 (14)	0.0189 (14)	0.0031 (11)	−0.0064 (11)	−0.0008 (11)
O4	0.0163 (13)	0.0249 (14)	0.0136 (13)	−0.0032 (11)	0.0028 (10)	−0.0062 (11)
O7	0.0244 (16)	0.0199 (15)	0.0401 (18)	−0.0023 (12)	−0.0165 (13)	0.0038 (13)
O1	0.0175 (14)	0.0368 (17)	0.0175 (14)	−0.0121 (12)	−0.0027 (11)	0.0062 (12)
O2	0.0178 (13)	0.0223 (14)	0.0114 (13)	−0.0066 (10)	−0.0004 (10)	0.0007 (10)
N1	0.0183 (16)	0.0247 (17)	0.0127 (15)	−0.0045 (13)	−0.0003 (12)	0.0010 (12)
N2	0.0151 (17)	0.0302 (19)	0.0146 (16)	−0.0066 (14)	0.0024 (13)	0.0016 (13)
C4	0.0174 (18)	0.0187 (18)	0.0090 (17)	0.0005 (14)	0.0004 (14)	−0.0010 (13)
C5	0.022 (2)	0.025 (2)	0.0090 (17)	−0.0024 (16)	0.0026 (14)	0.0013 (14)
C6	0.0124 (18)	0.0185 (19)	0.0153 (18)	−0.0013 (14)	0.0044 (14)	0.0015 (14)
C7	0.0126 (18)	0.024 (2)	0.0168 (19)	−0.0046 (15)	−0.0010 (14)	−0.0016 (15)
C3	0.0118 (17)	0.0191 (19)	0.0137 (18)	−0.0014 (14)	−0.0022 (14)	−0.0027 (14)
C2	0.0127 (17)	0.0126 (17)	0.0134 (17)	0.0004 (13)	0.0004 (14)	−0.0008 (13)
C8	0.0107 (17)	0.0172 (18)	0.0135 (18)	0.0037 (13)	−0.0006 (13)	−0.0003 (14)
C10	0.025 (2)	0.021 (2)	0.024 (2)	0.0016 (16)	0.0007 (17)	−0.0019 (16)
C1	0.0138 (18)	0.0101 (16)	0.0145 (18)	0.0017 (13)	−0.0010 (14)	−0.0013 (13)
C9	0.0136 (18)	0.0158 (18)	0.0127 (18)	−0.0054 (14)	0.0003 (14)	0.0019 (14)
C11	0.139 (7)	0.025 (3)	0.079 (5)	0.006 (3)	0.060 (5)	−0.014 (3)

### Geometric parameters (Å, º)

**Table:**

Ce1—O4	2.422 (2)	N2—C5	1.342 (5)
Ce1—O3	2.529 (3)	N2—C6	1.376 (4)
Ce1—O2^i^	2.543 (2)	N2—H2	0.857 (10)
Ce1—O5	2.547 (3)	C4—C3	1.388 (5)
Ce1—O1	2.568 (2)	C4—C6	1.392 (5)
Ce1—O7	2.580 (3)	C5—H5	0.9300
Ce1—O6	2.634 (3)	C6—C7	1.388 (5)
Ce1—N1	2.646 (3)	C7—C8	1.388 (5)
Ce1—O2	2.693 (2)	C7—H7	0.9300
Ce1—C10	2.960 (4)	C3—C2	1.394 (5)
Ce1—C1	3.002 (3)	C3—H3	0.9300
O6—C10	1.256 (5)	C2—C8	1.422 (5)
O5—C10	1.265 (5)	C2—C1^iii^	1.505 (5)
O3—C9	1.262 (4)	C8—C9^iv^	1.518 (5)
O4—C9^ii^	1.249 (4)	C10—C11	1.496 (6)
O7—H7B	0.830 (18)	C1—C2^iii^	1.505 (5)
O7—H7A	0.837 (18)	C9—O4^ii^	1.249 (4)
O1—C1	1.248 (4)	C9—C8^iv^	1.518 (5)
O2—C1	1.280 (4)	C11—H11A	0.9600
O2—Ce1^i^	2.543 (2)	C11—H11B	0.9600
N1—C5	1.306 (5)	C11—H11C	0.9600
N1—C4	1.403 (5)		
			
O4—Ce1—O3	80.07 (8)	C9—O3—Ce1	148.6 (2)
O4—Ce1—O2^i^	68.71 (8)	C9^ii^—O4—Ce1	131.1 (2)
O3—Ce1—O2^i^	70.91 (8)	Ce1—O7—H7B	124 (3)
O4—Ce1—O5	125.69 (8)	Ce1—O7—H7A	126 (3)
O3—Ce1—O5	134.99 (8)	H7B—O7—H7A	110 (3)
O2^i^—Ce1—O5	148.20 (8)	C1—O1—Ce1	97.7 (2)
O4—Ce1—O1	153.50 (9)	C1—O2—Ce1^i^	139.2 (2)
O3—Ce1—O1	74.17 (9)	C1—O2—Ce1	90.97 (19)
O2^i^—Ce1—O1	107.59 (8)	Ce1^i^—O2—Ce1	109.06 (8)
O5—Ce1—O1	71.89 (9)	C5—N1—C4	104.1 (3)
O4—Ce1—O7	97.53 (9)	C5—N1—Ce1	123.3 (2)
O3—Ce1—O7	144.86 (9)	C4—N1—Ce1	132.5 (2)
O2^i^—Ce1—O7	75.57 (9)	C5—N2—C6	107.0 (3)
O5—Ce1—O7	74.44 (9)	C5—N2—H2	127 (3)
O1—Ce1—O7	107.06 (9)	C6—N2—H2	126 (3)
O4—Ce1—O6	76.55 (8)	C3—C4—C6	120.0 (3)
O3—Ce1—O6	142.29 (8)	C3—C4—N1	130.5 (3)
O2^i^—Ce1—O6	125.00 (8)	C6—C4—N1	109.5 (3)
O5—Ce1—O6	50.21 (8)	N1—C5—N2	114.1 (3)
O1—Ce1—O6	121.56 (9)	N1—C5—H5	122.9
O7—Ce1—O6	68.14 (9)	N2—C5—H5	122.9
O4—Ce1—N1	84.39 (9)	N2—C6—C7	132.6 (3)
O3—Ce1—N1	76.65 (9)	N2—C6—C4	105.3 (3)
O2^i^—Ce1—N1	140.60 (9)	C7—C6—C4	122.0 (3)
O5—Ce1—N1	71.10 (9)	C8—C7—C6	118.1 (3)
O1—Ce1—N1	83.75 (9)	C8—C7—H7	121.0
O7—Ce1—N1	138.34 (10)	C6—C7—H7	121.0
O6—Ce1—N1	72.00 (9)	C4—C3—C2	119.4 (3)
O4—Ce1—O2	139.30 (8)	C4—C3—H3	120.3
O3—Ce1—O2	91.88 (8)	C2—C3—H3	120.3
O2^i^—Ce1—O2	70.94 (9)	C3—C2—C8	119.8 (3)
O5—Ce1—O2	87.74 (8)	C3—C2—C1^iii^	115.2 (3)
O1—Ce1—O2	49.17 (8)	C8—C2—C1^iii^	124.9 (3)
O7—Ce1—O2	67.05 (8)	C7—C8—C2	120.7 (3)
O6—Ce1—O2	124.89 (8)	C7—C8—C9^iv^	113.4 (3)
N1—Ce1—O2	132.72 (8)	C2—C8—C9^iv^	125.8 (3)
O4—Ce1—C10	101.21 (10)	O6—C10—O5	121.6 (4)
O3—Ce1—C10	146.18 (10)	O6—C10—C11	120.3 (4)
O2^i^—Ce1—C10	141.48 (9)	O5—C10—C11	118.1 (4)
O5—Ce1—C10	25.14 (10)	O6—C10—Ce1	62.8 (2)
O1—Ce1—C10	96.82 (10)	O5—C10—Ce1	58.8 (2)
O7—Ce1—C10	68.91 (10)	C11—C10—Ce1	176.0 (4)
O6—Ce1—C10	25.08 (9)	O1—C1—O2	120.3 (3)
N1—Ce1—C10	69.91 (10)	O1—C1—C2^iii^	118.3 (3)
O2—Ce1—C10	106.88 (10)	O2—C1—C2^iii^	121.4 (3)
O4—Ce1—C1	158.49 (9)	O1—C1—Ce1	57.99 (18)
O3—Ce1—C1	85.24 (9)	O2—C1—Ce1	63.79 (17)
O2^i^—Ce1—C1	91.70 (9)	C2^iii^—C1—Ce1	164.7 (2)
O5—Ce1—C1	75.70 (9)	O4^ii^—C9—O3	123.6 (3)
O1—Ce1—C1	24.32 (9)	O4^ii^—C9—C8^iv^	119.0 (3)
O7—Ce1—C1	85.50 (9)	O3—C9—C8^iv^	117.0 (3)
O6—Ce1—C1	123.78 (9)	C10—C11—H11A	109.5
N1—Ce1—C1	107.48 (9)	C10—C11—H11B	109.5
O2—Ce1—C1	25.24 (8)	H11A—C11—H11B	109.5
C10—Ce1—C1	99.75 (10)	C10—C11—H11C	109.5
C10—O6—Ce1	92.2 (2)	H11A—C11—H11C	109.5
C10—O5—Ce1	96.0 (2)	H11B—C11—H11C	109.5

Symmetry codes: (i) −*x*+1, −*y*+1, −*z*+2; (ii) −*x*, −*y*+1, −*z*+2; (iii) −*x*+1, −*y*+1, −*z*+1; (iv) −*x*, −*y*+1, −*z*+1.

### Hydrogen-bond geometry (Å, º)

**Table:**

*D*—H···*A*	*D*—H	H···*A*	*D*···*A*	*D*—H···*A*
N2—H2···O5^v^	0.86 (3)	1.93 (3)	2.716 (4)	153 (4)
O7—H7*A*···O3^i^	0.84 (4)	2.05 (4)	2.850 (4)	158 (3)
O7—H7*B*···O6^vi^	0.83 (3)	1.95 (3)	2.777 (4)	176 (6)

Symmetry codes: (i) −*x*+1, −*y*+1, −*z*+2; (v) *x*−1, *y*, *z*; (vi) −*x*+1, −*y*, −*z*+2.

## References

[bb1] Bruker (2005). *APEX2*, *SAINT* and *SADABS* Bruker AXS Inc., Madison, Wisconsin, USA.

[bb2] Farrugia, L. J. (1997). *J. Appl. Cryst.* **30**, 565.

[bb3] Gao, Q., Gao, W.-H., Zhang, C.-Y. & Xie, Y.-B. (2008). *Acta Cryst.* E**64**, m928. 10.1107/S1600536808017595 PMC2961800 21202783

[bb4] Sheldrick, G. M. (2008). *Acta Cryst.* A**64**, 112–122. 10.1107/S0108767307043930 18156677

[bb5] Song, W.-D., Wang, H., Hu, S.-W., Qin, P.-W. & Li, S.-J. (2009). *Acta Cryst.* E**65**, m701. 10.1107/S1600536809019680 PMC2969534 21583055

[bb6] Song, W.-D., Wang, H., Li, S.-J., Qin, P.-W. & Hu, S.-W. (2009). *Acta Cryst.* E**65**, m702. 10.1107/S1600536809019904 PMC2969642 21583056

[bb7] Spek, A. L. (2009). *Acta Cryst.* D**65**, 148–155. 10.1107/S090744490804362X PMC2631630 19171970

[bb8] Yao, Y.-L., Che, Y.-X. & Zheng, J.-M. (2008). *Cryst. Growth Des.* **8**, 2299–2306.

